# Early high-fat feeding improves histone modifications of skeletal muscle at middle-age in mice

**DOI:** 10.1186/s42826-020-00060-2

**Published:** 2020-08-08

**Authors:** Toshihiro Yoshie, Chiharu Saito, Fuminori Kawano

**Affiliations:** 1 Department of Sports and Health Science, Faculty of Human Health and Science, Matsumoto University, 2095-1 Niimura, Matsumoto City, Nagano 390-1295 Japan; 2 Graduate School of Health Sciences, Matsumoto University, 2095-1 Niimura, Matsumoto City, Nagano 390-1295 Japan

**Keywords:** Aging, Skeletal muscle, Epigenetics, Histone modification, Gene expression, Glucose tolerance

## Abstract

The purpose of the present study was to investigate how the effects of high-fat diet feeding on the skeletal muscle persisted during aging using mice. Post-weaned male mice were fed a high-fat diet between 1- and 3-mo-old followed by return to supply a normal diet until 13-mo-old. Monthly physical tests demonstrated that age-related glucose intolerance that was generally developed after 10-mo-old in the control mice was significantly improved in mice fed a high-fat diet. Interestingly, mRNA expressions of *Pdk4*, *Ucp3*, and *Zmynd17* were up-regulated by high-fat feeding and persisted in the tibialis anterior muscle until 13-mo-old. At *Pdk4* and *Ucp3* loci, enhanced distributions of active histone modifications were noted in the high-fat-fed mice at 13-mo-old. In contrast, age-related accumulation of histone variant H3.3 at these loci was suppressed. These results indicated that epigenetic modifications caused by early nutrition mediated the changes in skeletal muscle gene expression during aging.

## Background

For all individuals, the decline in skeletal muscle mass as a result of aging, is a primary contributor to the decline in physical performance (e.g., daily activities). In contrast, the progression of age-related diseases largely differs between individuals. The Dunedin study was based on aging in a population-representative 1972–1973 birth cohort of 1037 young adults followed from birth to age 38 y with 95% retention. When subjects were 38 y old, their physiologies were examined to test whether this young population would show evidence of individual variation in aging despite remaining free of age-related disease. Belsky et al. [1] recently reported that the Dunedin Study contained longitudinal data on 18 biomarkers established as risk factors or correlates of chronic disease and mortality. They analyzed within-individual longitudinal change in these 18 biomarkers across chronological ages 26 y, 32 y, and 38 y to quantify each study member’s rate of physiological deterioration. It was also reported that individuals before midlife who were aging more rapidly were less physically able, showed cognitive decline and brain aging, self-reported worse health, and looked older. Although these results indicate that the pace of aging differs in individuals, the mechanisms responsible for the individuality of aging are unknown.

Individual differences have also been reported in response to exercise training and the pathogenesis of lifestyle-related diseases. Resistance training-induced muscle hypertrophy was highly variable between individuals, who could be classified into high and low responders [2, 3]. Previous studies using twins demonstrated that the risk of lifestyle-related diseases was primarily dependent on the individual’s lifestyle [4, 5]. In an experiment using rodents, Nakamura et al. [6] reported that disuse atrophy was not induced in fast-twitch skeletal muscles of rats with endurance exercise training experience in early life. It was further demonstrated that a subset of genes that were generally upregulated in sedentary rats after muscle inactivation by tail suspension were less responsive in training-experienced rats. Incorporation of histone variant H3.3 was also promoted at these loci in rats with training experience. Ohsawa et al. [7] reported that exercise training-associated exchange of histone components from canonical histone 3 (H3) to H3.3 in skeletal muscle was closely related to the amount of daily exercise. These studies suggest that epigenetic remodeling is induced in association with lifestyle in early life, and persists through later life, leading to an altered response of gene transcription to physiological stimuli. However, nutrition-related histone modifications in skeletal muscle is poorly understood. For further understanding of individual differences in the physiological responses of skeletal muscle, the present study was carried out to investigate how the effects of high-fat diet feeding on the skeletal muscle persisted during aging using mice.

## Methods

### Experimental animals

Male C57BL/6 J mice (3-wk-old, *n* = 36) were purchased from CLEA Japan, Inc. and used in the present study. The mice were acclimated to the experimental environment followed by separating into the experimental groups, Control (*n* = 18) and High fat (*n* = 18). Temperature and humidity in the animal room were maintained at 23 °C and 40–60%, respectively, with 12:12 h light: dark cycle.

### Experimental design

Mice in High fat group were fed a commercial high-fat diet (High Fat Diet 32, 60% kcal, CLEA Japan, Inc.) between 1- and 3-month-old. Control diet (10% kcal, CLEA Japan, Inc.) was also supplied to mice in Control group. At the age of 3 months, the mice in both groups were returned to normal solid diet (CE-2, CLEA Japan, Inc.). A diet and water were supplied ad libitum. Tissue sampling was performed in 6 mice for each group at 3-mo-old. Remaining 12 mice in each group were tested physiological functions on every month through to 13-mo-old. Final tissue sampling was performed in 12 mice for each group at 13-mo-old.

### Physiological test

Hang test and intra-peritoneal glucose tolerance test (IPGTT) were performed to evaluate the age-associated changes of physiological functions. Neuromuscular strength was assessed using hang test. The mouse was placed on a wire mesh that was then inverted, and hung on a wire mesh by their four limbs. The latency to fall from the wire mesh was recorded with a 180 s cut-off time. In the IPGTT, mice were fastened for 20 h and fasting blood glucose levels in the tail vein were measured using LAB Gluco (ForaCare, Inc.). Glucose (2 mg/g body weight) in PBS was intraperitoneally injected and blood glucose levels were measured after 30, and 120 min. Successful glucose load was confirmed in the blood glucose level after 30 min, which data were not used for the evaluation of glucose tolerance. Physiological tests were performed through 3 days; on the 1st day hang test was performed in all mice (*n* = 12 in each group) followed by start fasting for half number of mice, on the 2nd day IPGTT was assessed in the first half number of mice and fasting was started for remaining half, and on the 3rd day IPGTT was assessed in these mice.

### Tissue sampling and muscle preparation

Tissue sampling was performed at 3-mo-old (*n* = 6 for each group) and 13-mo-old (*n* = 12 for each group) under anesthesia with *i.p.* injection of sodium pentobarbital (5 mg/100 g body weight). Left and right tibialis anterior muscles, triceps surae, and epididymal fat were sampled. The tibialis anterior muscle was frozen in liquid nitrogen-cooled isopentane and stored at − 80 °C until the analyses. Only weights were measured for triceps surae, and epididymal fat.

### Immunohistochemistry

Cross sections from the midportions of muscles were cut at 10 μm in a cryostat (Leica Microsystems) maintained at − 20 °C. The sections were fixed in 4% paraformaldehyde for 5 min, and degreased in methanol at − 20 °C for 10 min. Subsequently, antigen retrieval was performed in Antigen Retrieval Buffer (pH 6.0, Abcam) with heating using a microwave. The sections were permeabilized in PBS containing 1% triton X-100 for 10 min followed by blocking in Mouse Ig blocking reagent (Vector Laboratories) for 1 h. Pax7 (Mouse IgG, Developmental Studies Hybridoma Bank), dystrophin (Rabbit IgG, ab15277, Abcam) and type I myosin heavy chain (MyHC) (Mouse IgG, NCL-MHCs, Leica Microsystems) were labeled using specific antibodies diluted 1:100 in 0.1% triton X-100 (TPBS) containing 1% BSA. Labeling of dystrophin and type I MyHC was performed without antigen retrieval. Visualization for the binding site of primary antibody was performed using Alexa Fluor 488 and 594 (Molecular Probes) diluted 1:500 in TPBS containing 1% BSA for rabbit and mouse antibodies respectively, for 4 h. Stained sections were mounted using ProLong Diamond Antifade Mountant (Thermo Fisher Scientific) containing DAPI.

### Image analysis

The images of stained sections were analyzed using All-in-One Fluorescence Microscope BZ-X710 system (KEYENCE). Images of the portions in the section were computerized to construct the whole cross-section image. To analyze muscle fiber cross-sectional area (CSA), areas enclosed by dystrophin staining were selected within whole cross-section. To analyze the number of satellite cells, Pax7-positive cells were counted within whole cross-section. Approximately 2000 fibers or 5000 nuclei were analyzed in each section for analyzing fiber CSA or number of satellite cells, respectively. The number of satellite cells was expressed as the number per 1000 nuclei.

### RNA extraction

A piece of muscle (~ 20 mg) was homogenized in 1 mL ISOGEN (NIPPON GENE). RNA extraction was performed following the manufacturer’s instruction. The final pellet of RNA was resuspended in ultrapure water. For the gene expression analysis by quantitative PCR (qPCR), cDNA was synthesized from 800 ng total RNA using SuperScript VILO master mix (Invitrogen). The mixture of RNA was incubated at 42 °C for 60 min followed by the inactivation of enzyme at 85 °C for 5 min. cDNA was diluted at 1/100 by ultrapure water and stored at − 20 °C until the analyses.

### Chromatin immunoprecipitation (ChIP)

Muscle samples of the mice showed typical results in the gene expression analysis were selected (*n* = 3 for each group) for ChIP analysis. The procedure described previously [8] was modified and used for ChIP analysis. Briefly, a muscle segment (~ 40 mg) was homogenized in cooled PBS. After centrifugation, the pellet was fixed in 1% paraformaldehyde on ice for 10 min. Chromatin-rich extract was obtained by sonication using Sonifier 250 (Branson) followed by a gel filtration to remove small nucleotides and free histones. Chromatin was incubated with anti-total H3 (4620S, Cell Signaling Technology), anti-H3.3 (ab176840, Abcam), anti-pan-acetyl H3 (39,139, Active Motif), anti-H3 acetylated at lysine 27 (H3K27ac, 8173S, Cell Signaling Technology), anti-H3 tri-methylated at lysine 27 (H3K27me3, 9733S, Cell Signaling Technology), or anti-H3 mono-methylated at lysine 4 (H3K4me1, 5326S, Cell Signaling Technology) antibodies diluted 1:50 overnight at 4 °C, followed by subsequent reaction with protein G agarose beads (9007, Cell Signaling Technology; 20 μl for each reaction) for 4 h at 4 °C. Beads were washed and incubated with proteinase K (Takara Bio) for 1 h at 65 °C. DNA was extracted by adding phenol-chloroform solution (25:24) and by centrifugation at 12,000×g for 10 min at 20 °C. The supernatant was collected, and ethanol precipitation was performed using Ethachinmate (NIPPON GENE). The final pellet was resuspended in tris-EDTA buffer and stored at − 20 °C. The amount of input DNA in chromatin utilized for the ChIP reaction was estimated with the same procedure although without using any antibodies.

### qPCR

qPCR analysis was performed using StepOne Real Time PCR System (Thermo Fisher Scientific). The THUNDERBIRD qPCR Mix (TOYOBO) was used for PCR reaction following manufacturer-recommended dilution procedures. Genes that are reportedly related to glucose tolerance or type 2 diabetes in skeletal muscle of mice were targeted to analyze the regulation of gene expression [9–15]. Primer pairs for the gene expression analysis are shown in Table 1. Primer pairs for the ChIP analysis were designed at every 0.5 kbp sequences between 1.5 kbp upstream and 1.5 kbp downstream from the transcription start site of target genes (Table 2). Quantification of the results in qPCR was performed by normalizing cycle-to-threshold (Ct) of the target amplification with that of glyceraldehyde-3-phosphate dehydrogenase (*Gapdh*) gene as the internal control for the gene expression assay, or with Ct of the respective input DNA for ChIP-qPCR (% input). To quantify the distribution of histones at the locus, the values (% input) were further normalized to the median of each gene position was 1, prior to the values obtained from 6 positions were averaged within the locus.

**Table 1:** Sequences of primer pairs for gene expression analysis

Gene symbol	Forward primer	Reverse Primer
*Gapdh*	CAAGGACACTGAGCAAGAGA	GCCCCTCCTGTTATTATGGG
*Nox2*	GGCACACATTCACACTGACC	GCATTGTTCCTTTCCTGCAT
*Pdk4*	AGCTGGTGAAGAGCTGGTATATCC	TCTGGTCTTCTGGGCTCTTCTC
*Ppargc1a*	CGGAAATCATATCCAACCAG	TGAGGACCGCTAGCAAGTTTG
*Ucp3*	CTGCACCGCCAGATGAGTTT	ATCATGGCTTGAAATCGGACC
*Zmynd17*	TAGGGCTTAACAGGCACTGGTCCCC	TTCTTGTGCTTTCGCCGCCGTG
*Glut4*	ATGGCTGTCGCTGGTTTCTC	ACCCATGCCGACAATGAAGT
*Irs1*	TTGCTTGGCACAATGTAGAA	GAGGATCGTCAATAGCGTAAC

**Table 2:** Sequences of primer pairs for ChIP-qPCR analysis

Gene symbol	Distance from transcription start site	Forward primer	Reverse Primer
*Pdk4*	−1.5 to − 1.0 kbp	CATTAGGCAGACCCCCAGTA	CATGAGAGCTCACCCAGTCA
	−1.0 to −0.5 kbp	CAAAGGCTGCTTTTTGGAAG	TGTGGTGGAAAATCCCAGTT
	−0.5 to 0 kbp	TCCTAGCGACCTGGGATCTA	CTAGAAAGGCCTGGCACAGT
	0 to + 0.5 kbp	GAGCTGTTCTCCCGCTACAG	CAGTCGTCCTAGCCCTGAAC
	+ 0.5 to + 1.0 kbp	ACACACCCCGCTGATCTCT	AAGGAGACAGAGGCCTTTCC
	+ 1.0 to + 1.5 kbp	CGCTTCACCCACTACAGGTT	TTCAAAACCGTGTGTCCTCA
*Ucp3*	−1.5 to −1.0 kbp	AACTCCCAGCAGAGGTTTGA	TGCTTTGAGCTCAGTCTGGA
	−1.0 to −0.5 kbp	AGCATGTCCATGTCATGAGG	TAGCCTCTTGTGGCTGGTTT
	−0.5 to 0 kbp	CGTCTTCTCCTCTCCCCTCT	GGTTTAGCTTCCGTGACCTG
	0 to + 0.5 kbp	TACCCAACCTTGGCTAGACG	AAAACACGGGAGTTGTGAGG
	+ 0.5 to + 1.0 kbp	TGTGGGTACCCCAGTAGAGC	CCCAAGTTTTGAAGGAGTGC
	+ 1.0 to + 1.5 kbp	GGATTGACAGGGAAGCAAAA	TGTGTTCTGCTCTGCTTTGG
*Zmynd17*	−1.5 to −1.0 kbp	CTCTGCTTGCTTCCTTCCTC	ATGGTGGCTCACAACCATTT
	−1.0 to −0.5 kbp	GGGAATCCTTTGGAATCCTT	CTTCATCCCCTACTCCCACA
	−0.5 to 0 kbp	AGTGGGTCCTTTCCTTTGGT	CCAAAGTCAGGCAAGAGGAG
	0 to + 0.5 kbp	CCAGGTCCTGACTTGTCTCG	TAAGCCCTAGGCTTCCTTCC
	+ 0.5 to + 1.0 kbp	CTTCCCACCTTCATCCAAAA	ACCCTTCATCCCCAAAGTCT
	+ 1.0 to + 1.5 kbp	GGTCTGGGTCTGTGATAGCC	GCAGCACACCACTTGAAAGA

### Statistical analysis

**Fig. 1:**
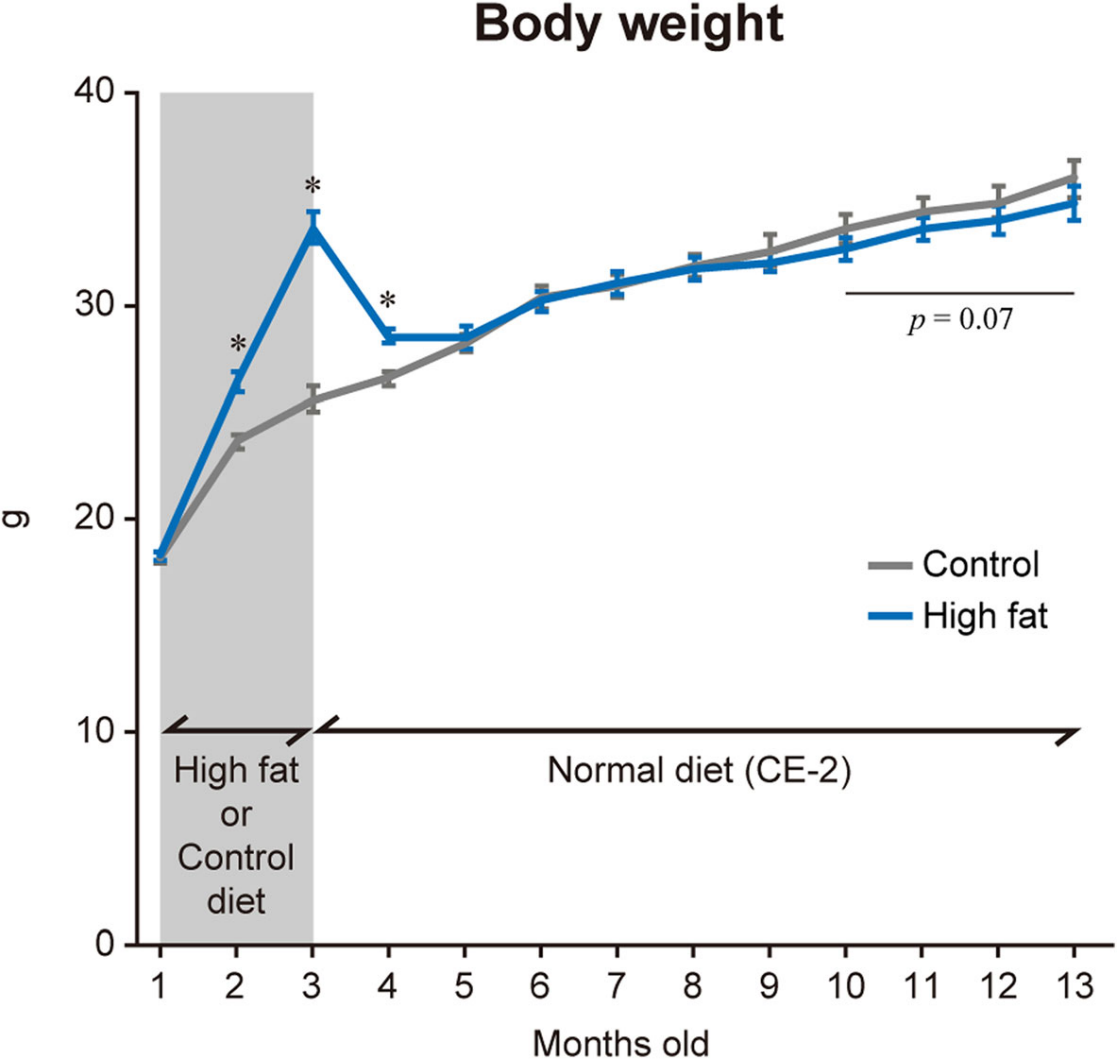
Time course change of body weight. Mice were separated into Control (gray) and High-fat (blue) groups, fed respective diet between 1- and 3-mo-old indicated by shaded area. Mean ± SE. *: *p* < 0.05 vs. age-matched control group examined by unpaired *t*-test

**Fig. 2:**
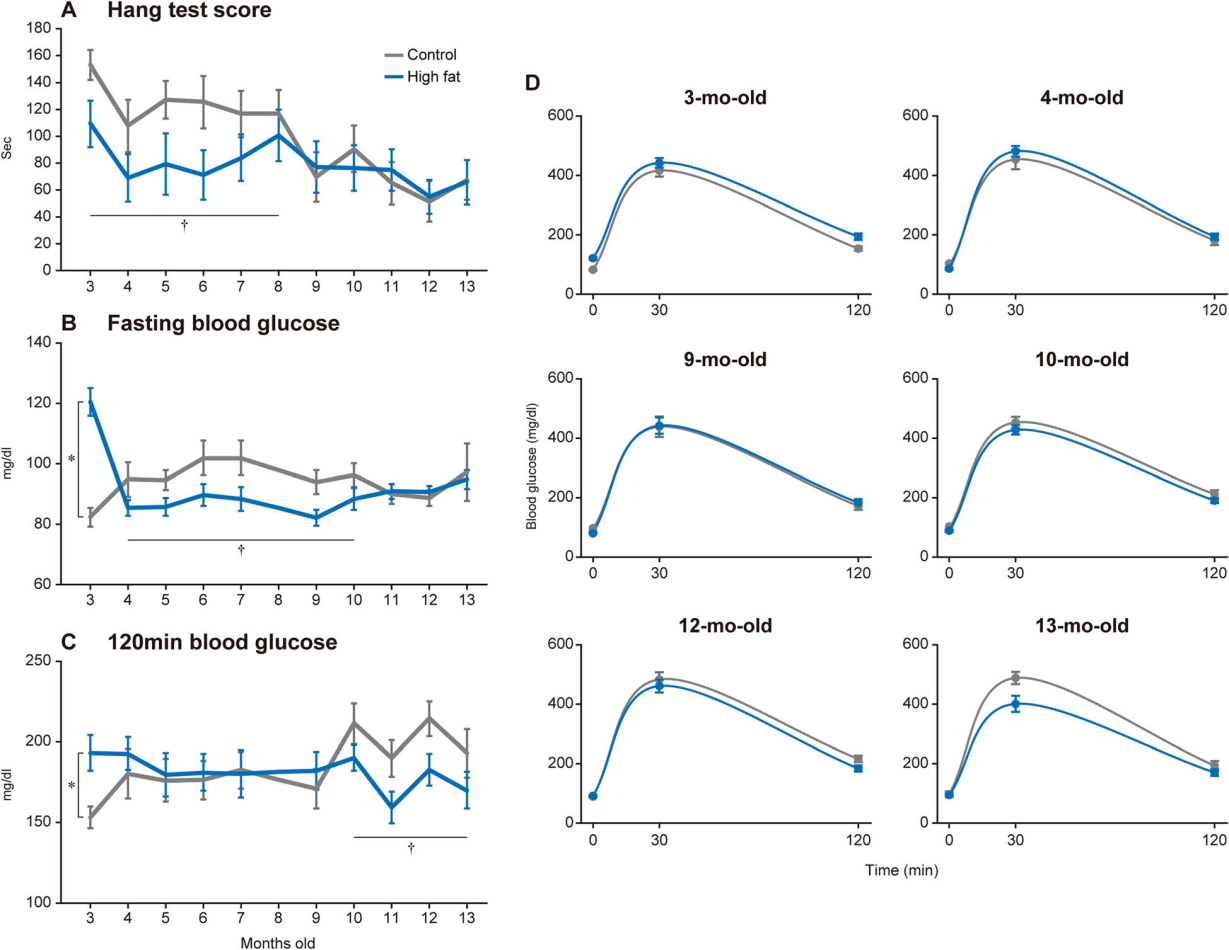
Results of physical test during aging. Monthly physical tests including hang test (**a**) and glucose tolerance test (**b** and **c**) was performed in both Control (gray) and High-fat (blue) groups (*n* = 12 in each group). **d** Time course changes of blood glucose during glucose tolerance test at typical ages. Values are shown as mean ± SEM. *, and †: *p* < 0.05 vs. age-matched control group examined by unpaired *t*-test, and by one-way ANOVA, respectively

Significant differences of the data were examined by unpaired *t*-test when the comparison was made between Control and High-fat groups at same age. To examine the sequent changes with age (Figs. 1 and 2), the maximum length of the continuous duration was selected by one-way ANOVA analysis if all values obtained from the duration were not statistically significant in the comparison of Control vs. High-fat groups. The same data sets were differently calculated and expressed in Figs. 4 and 5. To examine age-associated changes in histone modifications shown in Fig. 4, all % input data obtained in both Control and High-fat groups (*n* = 6 for each bar graph) were averaged in each locus, and 18 values obtained from all loci were used to compare between 3- and 13-mo-old by one-way ANOVA. In Fig. 5, the normalized values obtained from each position were averaged within the locus (6 positions per locus), because the levels of histone modifications were different by the position, and type of modification. Differences are considered significant at *p* < 0.05.

## Results

### Body and tissue weights

During high-fat diet feeding, body mass gain was significantly greater (+ 12.0% in 2-mo-old, and + 31.5% in 3-mo-old) in High-fat group compared to Control group (Fig. 1). After return to normal diet feeding, the body weight of High-fat group became identical to the level of Control group. Both High-fat and Control groups slightly increased the body weight through to 13-mo-old, but age-related gain of body weight tended to be less (− 3.3 to − 2.4% vs. Control group, *p* = 0.07) in High-fat group between 10- and 13-mo-old.

Epididymal fat weight was significantly greater in High-fat group (+ 123% and + 77% in absolute and relative weight, respectively) than that in Control group at 3-mo-old (total of left and right fats, Table 3). Both absolute and relative weight of epididymal fat were similar in both groups at 13-mo-old. Absolute weight of the tibialis anterior muscle was unchanged by high-fat feeding, but the weight relative to body weight was less (− 15% vs. Control group, *p* < 0.05) in High-fat group (mean of left and right muscles, Table 3). Absolute weight of triceps surae was greater (+ 8%) in High-fat than Control group at 3-mo-old, although the relative weight was lowered (− 14% vs. Control group) in High-fat group (*p* < 0.05) (mean of left and right muscles, Table 3). However, the skeletal muscle mass showed no differences between High-fat and Control groups at 13-mo-old.

**Table 3:** Changes of tissue weights after high-fat diet feeding

	3-mo-old		13-mo-old	
	Control	High-fat	Control	High-fat
Tibialis anterior				
Absolute weight (mg)	47.9 ± 0.9	50.9 ± 0.7	58.0 ± 0.5	57.2 ± 0.7
% body weight (%)	0.195 ± 0.026	0.165 ± 0.031*	0.164 ± 0.035	0.169 ± 0.030
Triceps surae				
Absolute weight (mg)	152.7 ± 1.8	165.5 ± 2.6*	186.4 ± 2.6	181.5 ± 1.8
% body weight (%)	0.623 ± 0.013	0.537 ± 0.093*	0.526 ± 0.114	0.535 ± 0.085
Epididymal fat				
Absolute weight (g)	0.55 ± 0.04	1.23 ± 0.06*	1.18 ± 0.13	1.16 ± 0.08
% body weight (%)	2.244 ± 0.137	3.964 ± 0.111*	3.244 ± 0.003	3.389 ± 0.002

Mean ± SE. *: *p* < 0.05 vs. age-matched Control group

### Physical test

Mean time to fall in the hang test was 153 and 109 s in Control and High-fat groups, respectively, at 3-mo-old (Fig. 2a). While the hang test score was gradually decreased with age in Control group, lowered hang test score in High-fat group was maintained until 8-mo-old (*p* < 0.05). However, no differences were observed in the hang test score between Control and High-fat groups after 8-mo-old.

Level of fasting blood glucose was 82.2 mg/dl in Control group at 3-mo-old, whereas high-fat diet feeding increased the level up to 120.7 mg/dl (*p* < 0.05, Fig. 2b). Blood glucose level in 120 min after glucose loading was also significantly increased by high-fat diet feeding (153.0 vs. 193.2 mg/dl in Control and High-fat groups, respectively) (Fig. 2c). These changes were normalized in 1 month after the end of high-fat feeding. The level of fasting blood glucose, in contrast, was maintained at low until 10-mo-old in High-fat group (*p* < 0.05, Fig. 2b). The fasting blood glucose in High-fat group was normalized to the level of Control group during further aging. Although 120-min blood glucose level was slightly increased (190.0 to 214.8 mg/dl) after 10-mo-old in Control group, that was significantly lowered (159.0 to 190.0 mg/dl) between 10- and 13-mo-old in High-fat group (Fig. 2c). The results of glucose tolerance tests at typical ages were shown in Fig. 2d. Although the deteriorated result of IPGTT was noted in High-fat group at 3-mo-old, that was completely normalized at 9-mo-old. At 12- and 13-mo-old, blood glucose level of Control group was more elevated in 30 and 120 min after glucose administration than that of High-fat group.

### Immunohistochemistry

Table 4 shows the results of immunohistochemical analysis. No significant differences were observed in the fiber size and the distribution of type I MyHC expressing fibers in the tibialis anterior muscle by high-fat diet feeding. Although the trend in the decrease of number of satellite cells was seen in 3- vs. 13-mo-old mice, the number was not affected by high-fat diet feeding at each time point.

**Table 4:** Histochemical characteristics of the tibialis anterior muscle

	3-mo-old		13-mo-old	
	Control	High-fat	Control	High-fat
Fiber cross-sectional area (μm^2^)	1981 ± 62	1865 ± 73	1840 ± 33	1858 ± 32
Type I fiber distribution (%)	ND	ND	ND	0.03 ± 0.0002
Number of Pax7-positive cells per 1000 nuclei	38.8 ± 5.0	43.2 ± 4.5	28.0 ± 1.6	28.9 ± 2.2

Mean ± SE

*ND* not detected

### Gene expression

**Fig. 3:**
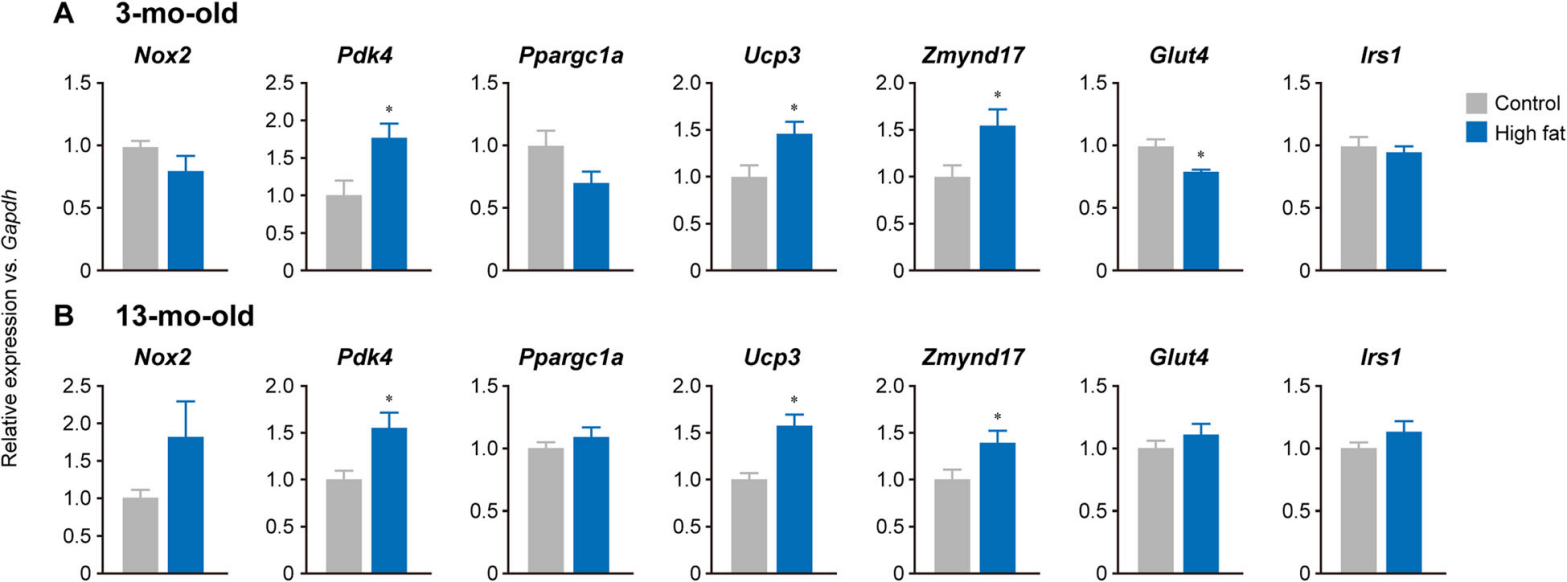
Effects of high-fat diet feeding on gene expressions in the tibialis anterior muscle. Gene expressions were measured in Control (gray) and High-fat (blue) groups at 3-mo-old (*n* = 6 in each group, **a**) and 13-mo-old (n = 12 in each group, **b**). All values of each gene obtained from qPCR were normalized with that of *Gapdh* mRNA, followed by the values were expressed to the mean of respective control level was 1. Values are shown as mean ± SEM. *: *p* < 0.05 vs. age-matched control group examined by unpaired *t*-test. *Nox2*, NADPH oxidase 2; *Pdk4*, pyruvate dehydrogenase kinase 4; *Ppargc1a*, peroxisome proliferator-activated receptor gamma coactivator 1-alpha; *Ucp3*, mitochondrial uncoupling protein 3; *Zmynd17*, zinc finger MYND domain-containing protein 17; *Glut4*, glucose transporter type 4; *Irs1*, insulin receptor substrate 1

Mean Ct of *Gapdh* mRNA expression was 17.0 ± 0.07 and 16.9 ± 0.09 (mean ± SD) at 3-mo-old, and 17.1 ± 0.09 and 16.9 ± 0.06 at 13-mo-old between Control and High-fat groups, respectively, indicating less variation in the expression of the reference gene. Expressions of pyruvate dehydrogenase kinase 4 (*Pdk4*), mitochondrial uncoupling protein 3 (*Ucp3*), and zinc finger MYND domain-containing protein 17 (*Zmynd17*) mRNA were significantly up-regulated (+ 78%, + 46%, and + 55% vs. Control group, respectively) in High-fat group at 3-mo-old, while glucose transporter type 4 (*Glut4*) mRNA level was significantly down-regulated (− 21% vs. Control group) (Fig. 3a). Up-regulated *Pdk4*, *Ucps3*, and *Zmynd17* mRNA expressions were maintained (+ 56%, + 57%, and + 39% vs. Control group, respectively, *p* < 0.05) until 13-mo-old in High-fat group, although *Glut4* mRNA level was normalized to the level of Control group (Fig. 3b).

### ChIP

**Fig. 4:**
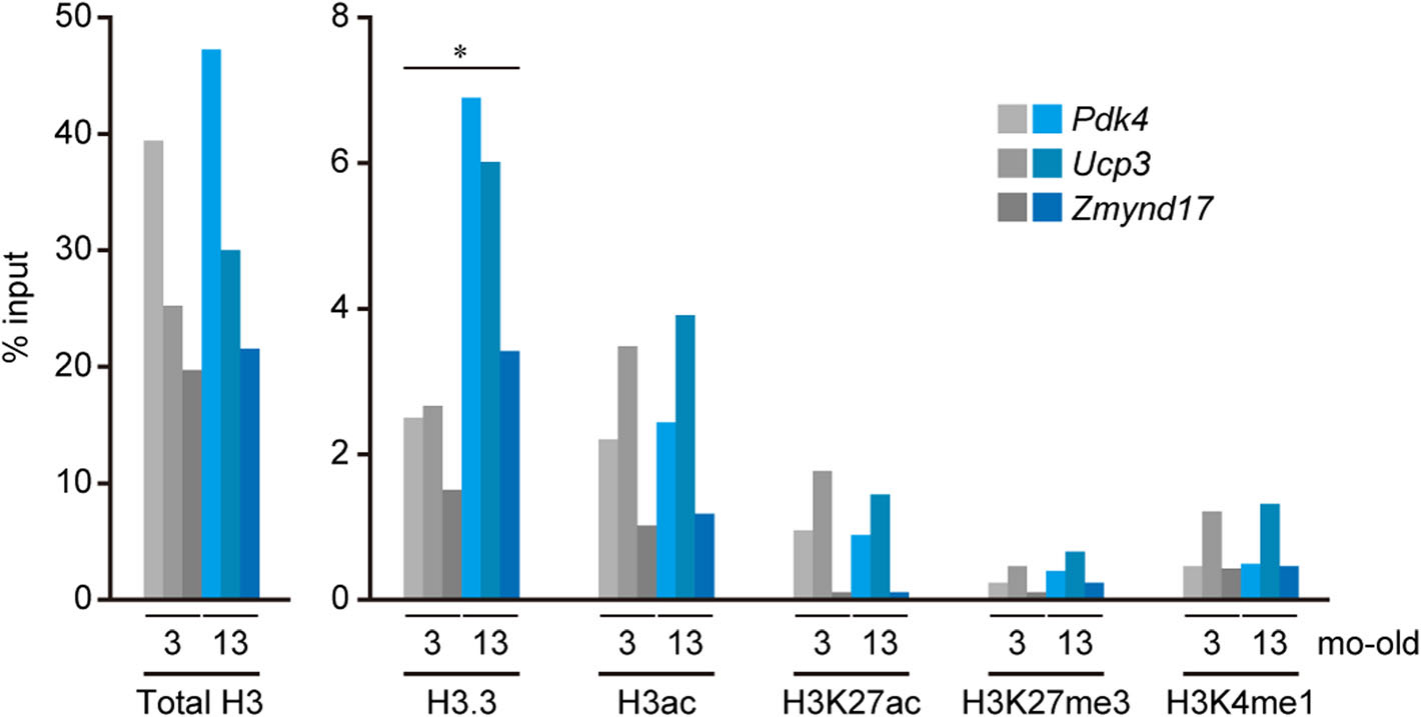
Age-related changes of histone distributions in the tibialis anterior muscle. ChIP-qPCR analysis was performed using 3 muscle samples selected in each group that showed the typical expression pattern in gene expression analysis. Distributions of total H3, H3.3, pan-acetyl H3 (H3ac), H3 acetylated at lysine 27 (H3K27ac), H3 tri-methylated at lysine 27 (H3K27me3), and H3 mono-methylated at lysine 4 (H3K4me1) were compared by the locus and the age. The values of ChIP results (% input) obtained from Control and High fat groups were combined (*n* = 6 for each bar graph), averaged, and compared between 3- and 13-mo-old. Note that distribution of H3.3 elevated with age in all loci. *: *p* < 0.05 in 3- vs. 13-mo-old examined by one-way ANOVA. *P*, *Pdk4*; *U*, *Ucp3*; *Z*, *Zmynd17*

Absolute levels (% input) of distribution of each histone modification that was averaged between Control and High-fat groups and plotted by genes were shown in Fig. 4. *Ucp3* locus was most modified by H3ac, H3K27ac, H3K27me3, and H3K4me1, whereas *Zmynd17* locus was less in the modifications. Distribution of H3.3 was significantly increased (+ 175%, + 125% and + 128% in *Pdk4*, *Ucp3* and *Zmynd17*, respectively) in all loci at 13-mo-old compared to that at 3-mo-old.

**Fig. 5:**
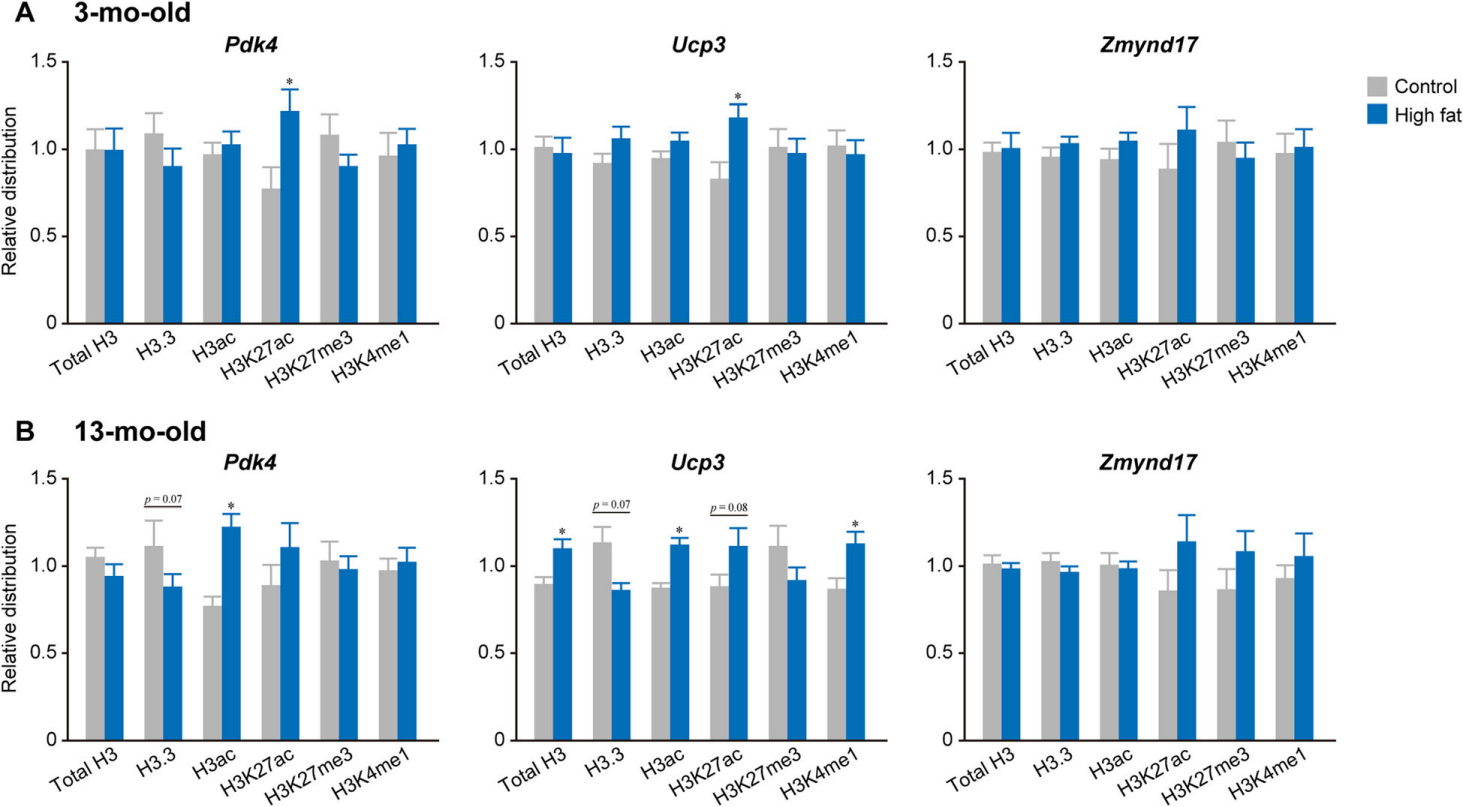
Effects of high-fat diet feeding on histone distributions in the tibialis anterior muscle. The same data sets used in Fig. 4 were differently analyzed to evaluate the effects of high fat diet feeding. Control (gray) and High-fat (blue) groups in 3-mo-old (**a**) and 13-mo-old (**b**). To normalize the differences in the distribution of data among genes or among type of modifications, the median value was calculated within each gene and modification, and expressed as 1. Values are shown as mean ± SEM. *: *p* < 0.05 vs. age-matched control group examined by unpaired *t*-test

It was noted that the distribution of H3K27ac increased at *Pdk4* and *Ucp3* loci after high-fat diet feeding at 3-mo-old (+ 58% and + 42% vs. Control group, respectively. *p* < 0.05), although no significant changes were observed in other histone modifications (Fig. 5a). *Zmynd17* locus did not show any significant changes in the modifications in response to the transcriptional activation by high-fat diet feeding. At *Pdk4* locus, the distributions of H3.3 (− 21%, *p* = 0.07) and H3ac (+ 60%, *p* < 0.05) were lowered and enhanced, respectively, in High-fat group compared to that in Control group at 13-mo-old (Fig. 5b). At *Ucp3* locus, the levels of total H3 (+ 23%, *p* < 0.05), H3ac (+ 28%, *p* < 0.05), H3K27ac (+ 26%, *p* = 0.08), and H3K4me1 (+ 30%, *p* < 0.05) distributions were greater in High-fat group compared to that in Control group at 13-mo-old, whereas that of H3.3 was less (− 24%, *p* = 0.07) in High-fat group (Fig. 5b). However, there were not significant changes in the histone modifications at *Zmynd17* locus in 13-mo-old (Fig. 5b).

## Discussion

### Locomotor disability

Age-associated decreases in locomotor functions were reported to develop by middle age in mice [16]. Latency to fall in the wire hang test was severely dropped in mice with the age between 8- and 12-mo compared to mice younger than 8-mo-old. In contrast, grip strength was not affected by aging [16]. It was suggested that age-associated decrease of locomotor functions was more pronounced in hindlimb muscles. The results of the present study showed a slight decrease in the hang test score after 8-mo-old in Control group (Fig. 2a), which agreed with the previous study [16]. However, although age-related body mass gain was seen in Fig. 1, relative weights of hindlimb muscles (Table 3), as well as fiber CSA of the tibialis anterior muscle (Table 4), were similar between 3- and 13-mo-old mice. These results suggested that failure in the neuro-motor tract, rather than loss of muscle mass, caused the locomotor disability in association with aging. High-fat feeding also deteriorated the hang test score in 3-mo-old mice, without changes in muscle mass and fiber size (Fig. 2a). Increased body weight was suggested to affect the hang test score. However, increased body weight was normalized within 2 months after the end of the high-fat diet feeding, suggesting that body mass gain was not a primary cause to induce locomotor disability.

Satellite cells are a postnatal source of skeletal muscle fibers. Previous studies demonstrated that the number of satellite cells declined with age, with 50% in old individuals compared to young and/or adult muscle [17–19]. Satellite cells within the aged niche poorly self-renewed and were less functional to differentiate into myonuclei, which occurred with a higher rate of cell death compared to satellite cells derived from the young niche [19]. Given that the present study did not show the significant effects of high-fat diet feeding on aging-associated changes in the number of satellite cells, suggesting that preserving satellite cells maintained muscle mass and locomotor function during post-high-fat-fed aging.

### Metabolic impairment

The results of epididymal fat weight clearly show the gain of visceral white adipose tissue volume after high fat diet feeding, but that is normalized at 13-mo-old (Table 3). It is known that adipose tissue expansion in obesity is mediated by hypertrophy (enlarged adipocytes), hyperplasia (increased numbers of adipocytes), or both [20, 21]. Jeffery et al. [22] tested high fat diet feeding in mice during early life period similar to the present study, reporting significantly increased formation of adipocytes exclusively in visceral white adipose tissue of high fat-fed mice, not in subcutaneous white adipose tissue. It was suggested that adipose tissue expansion during high fat diet feeding in the present study was also associated with hyperplasia of adipocytes. It was further speculated that normalized epididymal fat weight in High-fat group was related to enhanced energy metabolism. Slight inhibition of body mass gain after 10-mo-old in High-fat group may be related to the metabolic improvement, since hindlimb muscle weights were identical between Control and High-fat groups at 13-mo-old.

Previous studies reported that type 2 diabetes was induced by chronic high-fat feeding in mice, in which severe increases in fasting blood glucose and failure of glucose uptake to skeletal muscle were noted [11, 13, 14, 23]. These results of the glucose tolerance test indicated the successful induction of diabetes by a high-fat diet for 2 months (Fig. 2b). The results from gene expression analysis revealed an increase of *Pdk4*, *Ucp3*, and *Zmynd17* mRNA expression as well as a decrease in *Glut4* mRNA expression (Fig. 3). These changes regarding gene expression after a high-fat diet agreed with findings in previous studies [9–11, 13, 15]. Surprisingly, age-associated impairment of glucose tolerance that occurred after 10-mo-old in Control group was inhibited in High-fat group (Fig. 2b). Although abnormal glucose tolerance disappeared 1 month after ending high-fat feeding, up-regulated expression of *Pdk4*, *Ucp3*, and *Zmynd17* mRNA persisted in High-fat group until mice were 13-mo-old. Fujita et al. [11] reported that *Zmynd17* knockout mice showed an enhanced abnormality in glucose tolerance induced by high fat diet feeding. Choi et al. [10] reported that UCP3 overexpression in skeletal muscle protected mice from insulin resistance induced by high fat diet feeding. These results indicate that *Ucp3* and *Zmynd17* functioned to counteract the developing abnormal glucose tolerance. Therefore, it was suggested that continued expression of these genomic factors in skeletal muscle inhibited the decrease in glucose tolerance during aging. Furthermore, the normalization of *Glut4* mRNA expression supported our result that glucose tolerance in High-fat group was maintained, since GLUT4 protein plays a direct role for glucose uptake into skeletal muscle [9, 15].

NOX2 is one of the protein components of NADPH oxidase complex. Souto Padron de Figueiredo et al. [14] reported that knockout of *Nox2* improved the insulin resistance induced by high fat diet feeding via reducing the production of reactive oxygen species in skeletal muscle. They also showed the increased expression of *Nox2* mRNA after 3-mo high fat diet feeding in mice, although the expression of *Nox2* mRNA was unchanged in the present study. This difference might be due to the period assigned for high fat diet feeding. PPARGC1A, also known as PGC-1α, plays a crucial role for oxidative metabolism in skeletal muscle [24]. It was reported that muscle-specific PGC-1α overexpressing mice was more susceptible to high fat diet feeding [25, 26]. Although altered expression of *Ppargc1a* mRNA in skeletal muscle might be one of causes improve the glucose intolerance in middle-aged mice, the results of the present study showed no changes between Control and High fat groups at 13-mo-old. Insulin receptor substrate IRS1 mediates insulin-derived signal via phosphorylation, which activates its downstream effector, AKT, to cause GLUT4 translocation to the membrane [12]. According to the result showing no change in *Irs1* mRNA expression after high fat diet feeding, it was suggested that the level of gene expression was not related to glucose intolerance in skeletal muscle.

Interestingly, it is known that transcriptions of *Pdk4* and *Ucp3* mRNA are also stimulated by aerobic exercise [27, 28]. PDK4 regulates the activity of pyruvate dehydrogenase complex, which catalyzes the oxidative decarboxylation of pyruvate prior to entering tricarboxylic acid cycle during glucose oxidation. The expression of PDK4 is elevated when energy sources are switched from the utilization of glucose to fatty acids [29]. UCP3 is one of the uncoupling proteins that localize at inner mitochondrial membrane transporters and dissipates the mitochondrial proton gradient. UCP3 transgenic mice displayed a reduced fasting plasma glucose and insulin concentration [30]. These observations indicate that these factors enhance a capacity of mitochondrial oxidative phosphorylation in skeletal muscle. Therefore, it was suggested that in terms of lipid metabolism, high-fat diet feeding was similar to stimulation from aerobic exercise. Furthermore, the result showing that fasting blood glucose was maintained at lower levels in High-fat group for 6 months after the end of high-fat diet feeding might be related to enhanced lipid metabolism in skeletal muscle.

Genes up-regulated by high-fat diet feeding were shown to persist in later life, yet, this observation was not seen in the down-regulated gene. Enhanced active histone modifications by H3 acetylation and H3K4me1 caused the continuous expression of *Pdk4* and *Ucp3* mRNA in High-fat group at 13-mo-old. However, only H3K27ac was enhanced at the end of the high-fat diet feeding in High-fat group, which may be as a result of the correlated recruitment of RNA polymerase II during active transcription [31]. Although it is unclear how other histone modifications were added during the aging period, the experience of chronic high-fat diet feeding might alter the recruitment of histone modifiers at transcriptionally activated loci. A few modifications in absolute level compared by the values of % input (Fig. 4) were observed at *Zmynd17* locus in the tibialis anterior muscle of mice. The mechanisms responsible for a continuously enhanced *Zmynd17* mRNA expression in High-fat group at 13-mo-old were still unknown. However, it was speculated that loci with less prevalent histone modifications were less responsive to further modification.

Less incorporation of H3.3 at *Pdk4* and *Ucp3* loci was also pronounced in High-fat group of 13-mo-old mice, indicating that age-associated accumulation of H3.3 was suppressed by an early experience of high-fat diet feeding. This phenomenon might relate to the accumulation of active histone modifications at the loci. We previously reported that exercise training mediated the incorporation of H3.3 at the loci in the plantaris muscle of adult rats, which suppressed the gene expression in response to transcriptional activation by unloading [6], and that acetylated histones accumulated at the loci if H3.3 were less likely to be incorporated by exercise training [7]. These results also suggest that age-related H3.3 incorporation plays a critical role in subsequent modifications and gene responsiveness. Tvardovskiy et al. [32] demonstrated that a subset of histone methylations was accumulated on H3.3 of mouse liver, kidney, brain, and heart during aging, but modification patterns were distinct in each organ. Although they did not investigate this in skeletal muscle, it is suggested that H3.3-specific modifications are closely related to altered responsiveness of genes in skeletal muscle aging.

## Conclusions

In the present study, we have reported that using mice early experience of high-fat diet feeding affects the metabolic change during aging. Glucose tolerance deteriorated by high-fat feeding was normalized and maintained until 13-mo-old in high-fat-fed mice. In the tibialis anterior muscle, mRNA expressions of *Pdk4*, *Ucp3*, and *Zmynd17* were up-regulated by high-fat feeding and persisted until 13-mo-old. The differences of age-associated changes in the regulation of gene expression between control and high-fat-fed mice were supported by altered histone modifications, in which the enhanced distributions of active histone modifications, such as pan-acetylation and mono-methylation at lysine 4 of histone 3, were noted at *Pdk4* and *Ucp3* loci in the tibialis anterior muscle of high-fat-fed mice at 13-mo-old. These results indicated that epigenetic modifications caused by early nutrition mediated the changes in skeletal muscle gene expression during aging, resulting in altered metabolism in whole-body. The results also suggested that the nutritional environment in early life was one of causes for the individual differences in the pace of aging, might be partially due to the epigenetic changes in skeletal muscle.

## Data Availability

Not applicable.
